# The epigenetic clock is correlated with physical and cognitive fitness in the Lothian Birth Cohort 1936

**DOI:** 10.1093/ije/dyu277

**Published:** 2015-01-22

**Authors:** Riccardo E Marioni, Sonia Shah, Allan F McRae, Stuart J Ritchie, Graciela Muniz-Terrera, Sarah E Harris, Jude Gibson, Paul Redmond, Simon R Cox, Alison Pattie, Janie Corley, Adele Taylor, Lee Murphy, John M Starr, Steve Horvath, Peter M Visscher, Naomi R Wray, Ian J Deary

**Affiliations:** ^1^Centre for Cognitive Ageing and Cognitive Epidemiology, and; ^2^Centre for Genomic and Experimental Medicine, University of Edinburgh, Edinburgh, UK,; ^3^Queensland Brain Institute, and; ^4^Translational Research Institute, University of Queensland, Brisbane, QLD, Australia,; ^5^Department of Psychology, University of Edinburgh, Edinburgh, UK,; ^6^MRC Unit for Lifelong Health and Ageing, London, UK,; ^7^Wellcome Trust Clinical Research Facility, and; ^8^Alzheimer Scotland Dementia Research Centre, University of Edinburgh, Edinburgh, UK and; ^9^Gonda Research Center, David Geffen School of Medicine, Los Angeles, CA, USA

**Keywords:** epigenetic clock, fitness, longitudinal, DNA methylation, cognitive function

## Abstract

**Background:** The DNA methylation-based ‘epigenetic clock’ correlates strongly with chronological age, but it is currently unclear what drives individual differences. We examine cross-sectional and longitudinal associations between the epigenetic clock and four mortality-linked markers of physical and mental fitness: lung function, walking speed, grip strength and cognitive ability.

**Methods:** DNA methylation-based age acceleration (residuals of the epigenetic clock estimate regressed on chronological age) were estimated in the Lothian Birth Cohort 1936 at ages 70 (*n* = 920), 73 (*n* = 299) and 76 (*n* = 273) years. General cognitive ability, walking speed, lung function and grip strength were measured concurrently. Cross-sectional correlations between age acceleration and the fitness variables were calculated. Longitudinal change in the epigenetic clock estimates and the fitness variables were assessed via linear mixed models and latent growth curves. Epigenetic age acceleration at age 70 was used as a predictor of longitudinal change in fitness. Epigenome-wide association studies (EWASs) were conducted on the four fitness measures.

**Results:** Cross-sectional correlations were significant between greater age acceleration and poorer performance on the lung function, cognition and grip strength measures (r range: −0.07 to −0.05, P range: 9.7 x 10^−3^ to 0.024). All of the fitness variables declined over time but age acceleration did not correlate with subsequent change over 6 years. There were no EWAS hits for the fitness traits.

**Conclusions:** Markers of physical and mental fitness are associated with the epigenetic clock (lower abilities associated with age acceleration). However, age acceleration does not associate with decline in these measures, at least over a relatively short follow-up.

**Key Messages**

- DNA methylation age acceleration correlates cross-sectionally with physical and cognitive fitness in late life.
- DNA methylation age acceleration at age 70 does not predict decline in physical and cognitive fitness between the ages of 70 and 76 years.
- There is no correlation between the rate of change in DNA methylation age and the rate of change in physical or cognitive fitness.
- There are no epigenome-wide significant associations between individual CpGs and physical or cognitive fitness.

## Introduction

DNA methylation is an epigenetic marker that influences gene expression via the addition of a methyl group to cytosine nucleotides across the genome at cytosine-phosphate-guanine (CpG) sites.^1^ The proportion of methylation at individual CpG sites can be measured on a 0–1 scale, referred to as beta;^2^ these beta values are dynamic over time and can be influenced by both genes and the environment.^3^ The ‘epigenetic clock’ is a DNA methylation-derived measure that correlates highly with chronological age across the life course.^4–6^ We showed an association between the epigenetic clock and death, with a 21% increased mortality risk for those whose clock measure was 5 years above their chronological age.^7^

In that study,^7^ we did not examine markers of bodily fitness that might be related to epigenetic clock measures in later life. Furthermore, it is not generally known how changes in the epigenetic clock are influenced by or how they influence changes in such traits. One set of variables that may run in parallel with a biological clock are those that reflect fitness or general health and well-being.^8^ Markers of fitness include cognitive ability, grip strength, walking speed and lung function; lower levels in all of these measures are predictors of premature mortality.^9–16^

Here, we examine cross-sectional and longitudinal associations between the epigenetic clock and four measures of physical and mental fitness. We also examine, for the first time, longitudinal changes in the epigenetic clock in the same individuals. We hypothesize that the higher the baseline predicted age (epigenetic clock estimate) relative to chronological age, the poorer the score on the fitness traits cross-sectionally, and the greater the decline in the traits over time.

## Methods

### The Lothian Birth Cohort 1936

The Lothian Birth Cohort of 1936 (LBC1936) is a longitudinal study of ageing.^17^^,^^18^ Most of its members took part in the Scottish Mental Survey of 1947, when almost all children born in 1936 who attended school in Scotland on 4 June 1947 completed a test of general cognitive ability, the Moray House Test No. 12.^19^ Individuals born in 1936 who were living in the Lothian area of Scotland were contacted and invited to take part in LBC1936. In total, 1091 people were recruited at wave 1 (age ∼70 years) with further follow-up waves at ages ∼73 and ∼76. Extensive phenotypic data have been collected, including blood biomarkers, cognitive testing, and psycho-social, lifestyle, genetic and health measures.

#### Ethics

Ethical permission for the LBC1936 was obtained from the Multi-Centre Research Ethics Committee for Scotland (MREC/01/0/56) and the Lothian Research Ethics Committee (LREC/2003/2/29). Written informed consent was obtained from all subjects.

### LBC1936 DNA methylation

Details of DNA methylation measurement have been reported previously.^3^^,^^7^ Briefly, blood samples for methylation were taken from 1004 LBC1936 participants at wave 1, and sub-groups of 336 and 332 participants were sampled at waves 2 and 3, respectively. The decrease in sample size for these latter two waves was partly due to financial limitations rather than, for example, study dropout. DNA methylation typing was measured at 485 512 sites using the Illumina HumanMethylation450BeadChips. Extensive quality control was performed on these data, including the removal of probes with a low detection rate (<95% at P < 0.01). Low-quality samples, e.g. those with inadequate hybridization, bisulfite conversion, nucleotide extension or staining signal, were identified via manual inspection and removed. Samples with a call rate below 450 000 probes at P < 0.01 were removed. Similarly, samples where predicted sex, based on XY probes, did not match reported sex were removed, as were probes on the X and Y chromosomes. After these QC steps, 450 726 autosomal probes remained with 920, 299 and 273 samples available for analysis at wave 1 (age 70), wave 2 (age 73) and wave 3 (age 76), respectively. The background corrected probes were used to calculate DNA methylation age. For the epigenome-wide association studies, beta values were corrected for effects of sample plate, BeadChip, position on BeadChip and hybridization date using a generalized linear model with a logistic link function. Residuals from this model were used in further analyses.

### Data access

LBC data have been submitted to the European Genome-phenome Archive (EGA; https://www.ebi.ac.uk/ega/home) under accession number EGAS00001000910.

### Fitness Measures

Four fitness measures were considered: walking speed, grip strength, lung function and cognitive function.^20^ Walking speed was measured as the time to walk 6 m. Grip strength was assessed in the right hand three times using a North Coast Hydraulic Hand Dynamometer (JAMAR) with the best measure being carried forward. Lung function was measured as the forced expiratory volume in one second (FEV_1_) based on the highest score from three tests with a Micro Medical Spirometer. General fluid-type intelligence was derived from six non-verbal tests of cognitive function from the Wechsler Adult Intelligence Scale-III^UK^: letter-number sequencing and digit span backwards (working memory), matrix reasoning (non-verbal reasoning), block design (constructional ability), and digit symbol coding and symbol search (processing speed).^21^ The scores from the first unrotated component of a principal components analysis were extracted and labelled as general fluid ability, g*_f_*. This component explained 52% of the variance with individual test loadings ranging between 0.65 and 0.77.

### Covariates

In addition to age and sex, we adjusted for white blood cell counts in the epigenome-wide association study analyses.^22^^,^^23^ These included basophils, monocytes, lymphocytes, eosinophils and neutrophils that were measured on the same blood as that analysed for methylation. Details are reported in McIllhagger *et al.*^24^ Two additional covariates (height and smoking) were also included for some analyses. Smoking status was self-reported and categorized into never smoked, ex-smoker, and current smoker. Standing height was measured to the nearest millimetre using a SECA stadiometer.

### Statistical analyses

Methylation-based age acceleration was calculated for all subjects at each wave as the residuals from a linear regression model of methylation age on chronological age. Methylation age (epigenetic clock) estimates were calculated based on the algorithm of Horvath.^6^ The estimated age was calculated online (http://labs.genetics.ucla.edu/horvath/dnamage/), where background-corrected beta values were pre-processed using the calculator's internal normalization method. As the Horvath methylation age predictor is based on data from multiple tissue types, it is robust to differences in white blood cell counts (the numbers of basophils, monocytes, lymphocytes, eosinophils and neutrophils per volume of blood) taken from whole-blood samples.

Age- and sex-adjusted linear regression models with standardized fitness and age acceleration inputs were used to obtain the (semi-partial) correlations between the variables at wave 1 (age ∼70). Height was included as an additional covariate in the models for FEV_1_, grip strength and walking speed. Smoking status was included in the FEV_1_ model. Linear mixed models were used to test if either the fitness variables or the methylation age estimates (epigenetic clock measure, not age acceleration) changed over time. Covariates included age (centred to the minimum value (67.7 years)and used as the time scale; age convergence was assumed in the model) and sex. As with the cross-sectional associations, height was included in the models for FEV_1_, grip strength and walking speed; smoking status was included in the model for FEV_1_.Wave 1 age acceleration was then added as a fixed effect interaction with age to test if it predicted decline in the fitness measures.

Next, a series of bivariate latent growth curve models were used to test whether the slopes of later-life decline in the fitness variables were correlated with the slope of the increase in methylation age between age 70 (wave 1) and age 76 (wave 3).^25^ For these models, we used full-information maximum likelihood modelling to take into account all of the available data.

Finally, to determine whether individual methylation probes were associated with the fitness markers, epigenome-wide association studies were conducted using all available information across the three waves for each trait. The CpG probes were entered as the dependent variables; covariates included age, sex, white blood cell counts and the predictor of interest (cognition, walk speed, grip strength or lung function). Height was adjusted for in the FEV_1_, grip strength and walking speed analysis; smoking status was controlled for in the FEV_1_ model. An epigenome-wide significance threshold was set at 1.1 x 10^−7^ (0.05 divided by the total number of probes, 450 726).

Linear mixed models were run in R using the libraries ‘lme4’ and ‘lmerTest’, and the ‘lm’ function in the ‘stats’ library.^26–28^ Latent growth curve models were run in MPlus version 7.2.^29^

## Results

### Cohort information

**Figure 1.**
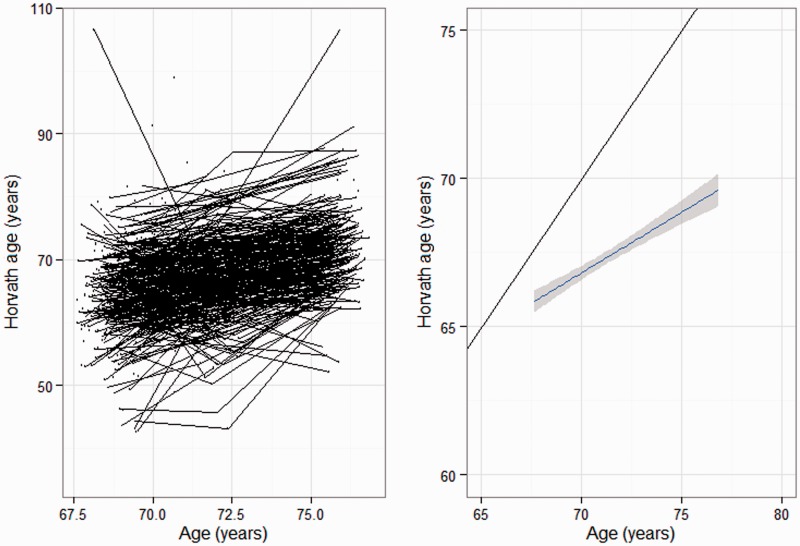
Individual trajectories and mean rate of change in DNA methylation age over time.

Details of the LBC1936 cohort are presented in Table 1. Of the 1091 subjects included in the cohort, the mean age at baseline was 69.5 [standard deviation (SD) 0.8] years, and 49.8% were female. The mean DNA methylation age was 65.9 (SD 6.5) years. At waves 2 and 3, the mean ages were 72.5 (SD 0.7) and 76.2 (SD 0.7) years, and the mean DNA methylation ages were 67.2 (SD 6.7) and 71.5 (SD 6.2) years, respectively. Individual trajectories and the mean rate of change in DNA methylation age are plotted in Figure 1. The fitness variables are also presented in Table 1 across the three waves. The mean fluid-intelligence cognitive score at baseline was 0.05 (SD 1.01), which declined at waves 2 (mean 0.03) and 3 (mean −0.07). Decline was also observed for grip strength and lung function across the three waves. Walking speed decreased over the waves, taking 3.9 s to walk 6 m at wave 1 compared with 4.7 s at wave 3.Compared with the participants with methylation data who completed more than one wave, those with methylation data from the first time point were slightly older with lower cognitive scores, poorer fitness scores and a higher methylation age (Supplementary Table 1, available as Supplementary data at *IJE* online).

**Table 1.** Summary of the Lothian Birth Cohort 1936 at waves 1 (age ∼70), 2 (age ∼73) and 3 (age ∼76)

	Wave 1			Wave 2			Wave 3		
	n	Mean	SD	n	Mean	SD	n	Mean	SD
Age (years)	1091	69.5	0.83	866	72.5	0.71	697	76.2	0.68
g*_f_*^a^	1072	0.05	1.01	856	0.03	0.97	668	−0.07	0.98
Grip strength (kg)	1085	29.0	10.1	864	28.6	9.5	693	27.7	10.1
FEV_1_ (l)^b^	1085	2.36	0.69	856	2.30	0.68	690	2.11	0.64
6-m walk time (s)	1085	3.86	1.16	860	4.35	1.32	693	4.71	1.72
Methylation age (years)^c^	920	65.9	6.5	299	67.2	6.7	273	71.5	6.2

		*n*	%		*n*	%		*n*	%

Sex (female)		543	49.8		418	48.3		337	48.4

SD, standard error.

^a^Fluid type general intelligence.

^b^Forced expiratory volume in one second.

^c^Epigenetic clock estimate of DNA methylation age.

### Cross-sectional correlations between methylation age acceleration and fitness

Table 2 presents the associations between age acceleration at wave 1 and the fitness variables. The linear regression coefficients are standardized betas after adjustment for age, sex and, where specified, height and smoking. These estimates are equivalent to semi-partial correlations. A higher age acceleration was significantly associated with lower cognitive scores (beta = −0.07, P = 0.024), weaker grip strength (beta = −0.05, P = 0.0097) and poorer lung function (beta = −0.06, P = 0.0064) at wave 1. Walking speed had a non-significant association with age acceleration (beta = 0.03, P = 0.45).

**Table 2.** Associations between age acceleration at wave 1 and fitness variables adjusted for age and sex

	Age acceleration		
	Beta^a^	SE	*P*
g*_f_*^b^	−0.07	0.03	0.024
Grip strength (kg)	−0.05	0.02	9.7 x 10^−3^
FEV_1_ (l)^c^	−0.06	0.02	6.4 x 10^−3^
6 -m walk time (s)	0.03	0.03	0.45

SE, standard error.

^a^Both age acceleration and the system integrity variables were standardized inputs to the regression analyses, implying that the beta values can be read as semi-partial correlations. Additional adjustments were also made for height (grip strength, FEV_1_ and 6-m walk) and smoking (FEV_1_).

^b^Fluid type general intelligence.

^c^Forced expiratory volume in one second.

### Longitudinal change in methylation age and fitness

Linear mixed model output for decline in the fitness traits and change in methylation age over time is presented in Table 3. There was strong evidence for decline in all fitness traits over the 6 years of follow-up, conditional on covariates. Cognition declined at 0.05 SDs per year, grip strength at 0.03 SDs per year, FEV_1_ at 0.07 SDs per year and walking speed at 0.10 SDs per year (all P < 2 x 10^−16^). Methlyation age increased at 0.14 SDs per year (P < 2 x 10^−16^), which corresponds to 0.91 years per year. There was no evidence of DNA methylation (DNAm) age acceleration changing over time: estimate 0.01 [standard error (SE) 0.008] SDs per year. There was also no evidence to suggest variance differences for DNAm age across the three waves (Bartlett's K^2^ = 2.1, df = 2, P = 0.35).

**Table 3.** Change in methylation age and fitness variables over time

	Beta^a^	SE	*P*
Methylation age(years)	0.14	0.007	<2 x 10^−16^
g*_f_*^b^	−0.05	0.003	<2 x 10^−16^
Grip strength (kg)	−0.03	0.003	<2 x 10^−16^
FEV_1_ (l)^c^	−0.07	0.002	<2 x 10^−16^
6-m walk time (s)	0.10	0.005	<2 x 10^−16^

^a^Beta values represent the change per SD in the dependent variable per year of ageing (centred as time scale) from a linear mixed model adjusting for sex with a random intercept. Additional adjustments were also made for height (grip strength, FEV_1_ and 6-m walk) and smoking (FEV_1_).

^b^Fluid type general intelligence.

^c^Forced expiratory volume in one second.

### Prediction of change in fitness based on baseline methylation age acceleration

There was no evidence to suggest that wave 1age acceleration was predictive of subsequent decline in any of the four fitness measures (Table 4). A weak association was seen for lung function, although the effect size was very small with a nominal P-value (standardized beta =7.8 x 10^−4^, P = 0.05), corresponding to an increase of 0.0005 l of FEV_1_ per year for each additional year of age acceleration. Using a linear regression model with data from waves 1 and 3 (ages 70 and 76 years), we estimated the proportion of variance in change in fitness explained by baseline age acceleration. For decline in lung function, 0.33% of the variance was explained whereas less than 0.06% was explained for the other traits.

**Table 4.** Effect of baseline age acceleration on longitudinal change in the fitness variables

	Baseline age acceleration		
	Beta^a^	SE	*P*
g*_f_*^b^	−4.4 x 10^−5^	4.6 x 10^−4^	0.92
Grip strength (kg)	9.2 x 10^−5^	4.5 x 10^−4^	0.84
FEV_1_ (l)^c^	7.8 x 10^−4^	4.0 x 10^−4^	0.05
6-m walk time (s)	5.6 x 10^−4^	8.1 x 10^−4^	0.49

SE, standard error.

^a^Beta values represent the change per SD in the dependent variable per additional year of age acceleration per year of follow-up from a linear mixed model adjusting for sex with a random intercept. Additional adjustments were also made for height (grip strength, FEV_1_ and 6 -m walk) and smoking (FEV_1_).

^b^Fluid type general intelligence.

^c^Forced expiratory volume in one second.

### Bivariate latent growth models of change in methylation age and fitness

The four bivariate latent growth curves had adequate-to-good fit, according to absolute fit indices (all root mean square error of approximation values <0.082; all comparative fit indices >0.925; all Tucker-Lewis indices >0.915). There was no evidence for coupled change in methylation age and general intelligence, FEV_1_, walking speed or grip strength: the effect sizes for the slope-slope correlations in the growth curve models were all non-significant (Table 5). Note that the standard error estimate for the coupled change in intelligence was very high, indicating an imprecise estimate.

**Table 5.** Associations of the slope of change in methylation age and the slope of change in system integrity variables between ages 70 and 76 years

	Methylation age slope		
	Beta^a^	SE	*P*
g*_f_*^b^ slope	−0.56	0.82	0.49
Grip strength (kg) slope	−0.08	0.38	0.83
FEV_1_ (l)^c^ slope	−0.20	0.15	0.18
6-m walk time (s) slope	0.16	0.26	0.55

SE, standard error.

^a^Betas represent the standardized slope-slope correlation from bivariate latent growth curve models.

^b^Fluid type general intelligence.

^c^Forced expiratory volume in one second.

### Epigenome-wide association studies of the fitness variables

**Figure 2.**
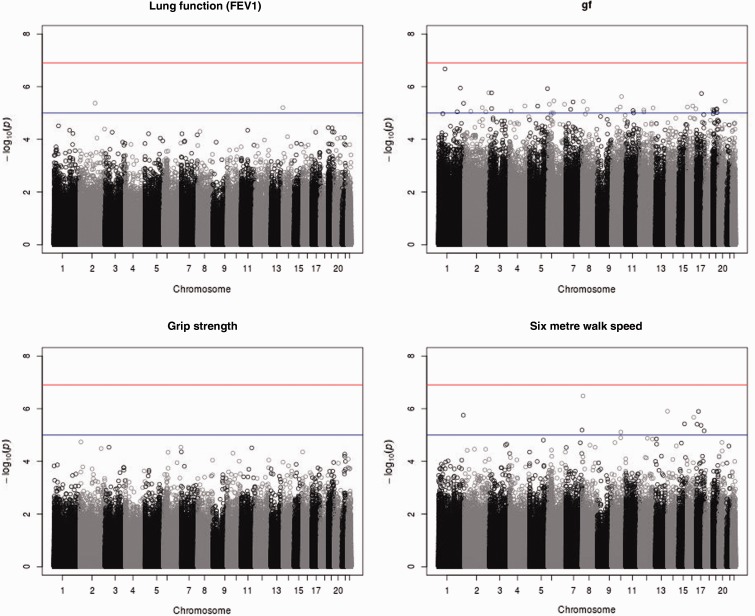
Epigenome-wide association study Manhattan plots for the four fitness traits. The lines indicate the Bonferroni significant P-value threshold (P = 0.05/450,726 = 1.1 x 10^−7^) and nominal significance (P = 1 x 10^−5^). g*_f_*, fluid type general intelligence; FEV_1_, forced expiratory volume in one second.

Manhattan plots from the epigenome-wide association studies (EWAS) analyses are shown for the four fitness traits in Figure 2. No probes passed the Bonferroni significance threshold for any of the traits. A list of suggestive hits at the P < 1 x 10^−5^ significance threshold are listed in Supplementary Table 2 (available as Supplementary data at *IJE* online).

## Discussion

In a cohort of 1091 older individuals followed up from ages 70 to 76, there was evidence to link DNA methylation-based age acceleration with measures of physical and mental fitness. We found significant cross-sectional correlations at age about 70 years between age acceleration and fluid cognitive ability, grip strength and lung function; higher methylation age acceleration was linked to poorer fitness. The four fitness variables showed modest rates of decline over the follow-up period, and methylation age increased at the same rate as chronological age. Wave 1 (age ∼70) age acceleration did not predict rate of change for any of the fitness variables. The methylation age and the fitness variables did not show correlated changes between ages 70 and 76.

There were no significant associations between individual CpG methylation sites and any of the four fitness traits. In a previous study on the 27 k methylation array, Bell *et al*.^31^ found no EWAS hits for grip strength but a solitary hit (cg16463460) for lung function. This hit did not replicate in LBC1936 (P = 0.60).

A strength of the study is the availability of longitudinal measures of fitness and DNA methylation on a moderately large sample with a narrow age range. However, not all participants had DNA methylation at waves 2 and 3, and so there was limited statistical power to test the associations between changes in methylation age and changes in fitness. For example, with a sample size of 300, the power to detect a correlation of 0.10 is 0.41 compared with 0.85 when the sample size is 900. It will be of interest to see how methylation age trajectories present in other large studies with data from across the life course. For example, do methylation age and age acceleration change linearly over time or is there a period in life with greater acceleration? Whereas the fitness measures are strong predictors of health outcomes and mortality,^9–16^ three are based on physical performance measures, with the other measuring complex cognitive ability. We did combine information from these four domains into a general factor (49% of the variance was explained and each test loaded strongly on the factor), which correlated −0.09 with methylation age (P = 3.8 x 10^−4^), declined over time, but was not predicted by baseline methylation age acceleration (P = 0.12). In addition, the ideal tissues to measure methylation directly related to these outcomes would be muscle (grip strength, walking speed), lung (FEV_1_), and brain (cognition). However, given that the methylation age predictor was derived from multiple tissue types, we argue that blood is a reasonable proxy measure for this analysis. We also ran the analysis using the blood-based Hannum predictor^5^ which yielded similar but weaker cross-sectional associations between methylation age acceleration and fitness (Supplementary Table 3, available as Supplementary data at *IJE* online) but no longitudinal findings. Future studies could consider longitudinal changes in biomarkers that might offer more information about mechanisms such as inflammatory and metabolic measures, e.g., an index of allostatic load.^32^ Other age-related biomarkers, such as telomere length, could also be considered.^33^^,^^34^

A previous study of age acceleration using data from four cohorts, including LBC1936, found no association between the measure and several health-related, genetic or lifestyle outcomes such as smoking, cardiovascular disease, diabetes, hypertension, smoking status and *APOE* e4 status.^7^ Here we provide some evidence to suggest that age acceleration may also be a marker of general physical and cognitive fitness in older individuals. Replication is required to determine whether the findings are consistent and, if so, the size of these associations, although the present study indicates that they are likely to be small. In contrast to the cross-sectional findings, there was no sign of wave 1 age acceleration predicting decline in fitness, or changing in concert with any of the fitness or cognitive variables. There are several explanations for these null results: (i) there are genuinely no longitudinal associations;(ii) the rate of change in the fitness variables is small (maximum decline 0.07 SDs per year), which means larger samples or longer follow-up periods may be required to identify effects; or (iii) related to the previous point, the cohort under investigation may be too young and fit to observe sufficiently large and reliable declines. In the present study, we find that the Horvath predictor slightly underestimates the ages (by about 4 years) in older subjects. However, this systematic deviation does not affect our measure of age acceleration which is defined as a residual from a linear regression model. Moreover, with a birth cohort, a bias in the intercept is of relatively minor importance as we are comparing high and low predicted ages for individuals who have essentially the same chronological age.

In conclusion, there is evidence to indicate an association between DNA methylation age and general bodily fitness as measured by four markers of physical and cognitive ability. Given the relatively modest rates of decline in what is a generally healthy sample of older people, it is perhaps not surprising that baseline levels of the epigenetic clock did not predict or correlate with rates of change in fitness. Following cohorts such as LBC1936 into older age and jointly modelling dropout due to mortality with decline in the fitness variables will provide a better understanding of the epigenetic clock as a biomarker of ageing.

## Supplementary Data

Supplementary data are available at *IJE* online.

## Funding

Phenotype collection in the Lothian Birth Cohort 1921 was supported by the UK’s Biotechnology and Biological Sciences Research Council (BBSRC), the Royal Society and the Chief Scientist Office of the Scottish Government. Phenotype collection in the Lothian Birth Cohort 1936 was supported by Age UK (the Disconnected Mind project). Methylation typing was supported by the Centre for Cognitive Ageing and Cognitive Epidemiology (Pilot Fund award), Age UK, the Wellcome Trust Institutional Strategic Support Fund, the University of Edinburgh and the University of Queensland. R.E.M., S.J.R., S.E.H., S.R.C., P.M.V., J.M.S. and I.J.D. are members of the University of Edinburgh Centre for Cognitive Ageing and Cognitive Epidemiology (CCACE). CCACE is supported by funding from the BBSRC, the Economic and Social Research Council (ESRC), the Medical Research Council (MRC) and the University of Edinburgh as part of the cross-council Lifelong Health and Wellbeing initiative (MR/K026992/1). Research reported in this publication was supported by National Health and Medical Research Council (NHMRC) [project grants 613608, APP496667, APP1010374 and APP1046880], NHMRC Fellowships to P.M.V. and N.R.W. [613602] and an Australia Research Council (ARC) Future Fellowship to N.R.W. [FT0991360)] The content is solely the responsibility of the authors and does not necessarily represent the official views of the NHMRC or ARC. G.M.T. is supported by NIH/NIA [1P01AG043362].

## Supplementary Material

Supplementary Data
