# Supplementary material for: The epigenetic clock is correlated with physical and cognitive fitness in the Lothian Birth Cohort 1936
Source: Int J Epidemiol. 2015 Jan 22;44(4):1388–96. doi: 10.1093/ije/dyu277 (PMC4588858; doi:10.1093/ije/dyu277)
Supplement: Supplementary Data [file supp_44_4_1388__index.html]

Supplementary Data 

# The epigenetic clock is correlated with physical and cognitive fitness in the Lothian Birth Cohort 1936

## Supplementary Data

files

- Supplementary Data - pdf file
