# Supplementary material for: The epigenetic clock is correlated with physical and cognitive fitness in the Lothian Birth Cohort 1936
Source: Int J Epidemiol. 2015 Jan 22;44(4):1388–96. doi: 10.1093/ije/dyu277 (PMC4588858; doi:10.1093/ije/dyu277)
Supplement: Supplementary Data [file supp_dyu277_ije-2014-10-1135.pdf]

Supplementary Table 1: Comparison of baseline data for participants with methylation data who were included at waves 2 and 3 versus those included only at wave 1.

	Wave 1 only			Wave 1, plus Wave 2 or 3		
	n	Mean	SD	n	Mean	SD
Age (years)	623	69.8	0.78	297	72.5	0.57
g_f*	611	-0.11	0.99	293	0.03	1.01
Grip Strength (kg)	620	28.4	10.2	296	28.6	10.1
FEV_1 (l)†	622	2.30	0.68	296	2.30	0.68
6m Walk Time (s)	620	3.94	1.31	296	4.35	0.93
Methylation age (years)‡	623	66.5	6.3	297	67.2	6.9
		N	%		N	%
Sex (female)		314	50.4		141	47.5

*fluid type general intelligence †Forced Expiratory Volume in one second ‡ Epigenetic clock estimate of DNA methylation age

Supplementary Table 2: Top hits at $P<1x10^{-5}$ from the epigenome-wide association study analysis.

Lung Function (FEV1)

CpG	Beta	SE	P	CHR	Position	Gene Name
cg14961391	-1.5E-01	3.2E-02	4.2E-06	2	148758708	origin recognition complex, subunit 4
cg23710823	-4.5E-02	9.8E-03	6.2E-06	14	20020657	POTE ankyrin domain family, member M

Gf

CpG	Beta	SE	P	CHR	Position	Gene Name
cg10316635	-8.3E-02	1.6E-02	2.1E-07	1	62208645	InaD-like (Drosophila)
cg18789663	-5.5E-02	1.2E-02	4.3E-06	1	242688591	phospholipase D family, member 5
cg23217419	-3.7E-02	8.3E-03	8.9E-06	1	180881088	KIAA1614
cg24086592	3.6E-02	7.2E-03	1.1E-06	1	211655732	retinal degeneration 3
cg01365117	3.5E-02	7.8E-03	8.9E-06	2	208761957	pleckstrin homology domain containing, family M, member 3
cg02091526	-4.8E-02	1.0E-02	6.2E-06	2	175190867	NA
cg11536474	-9.6E-02	2.1E-02	8.6E-06	2	63286049	NA
cg21963920	3.4E-02	7.0E-03	1.7E-06	2	237216266	NA
cg02252323	-5.4E-02	1.1E-02	1.7E-06	3	22414339	NA
cg25839023	-5.2E-02	1.1E-02	6.8E-06	3	22414349	NA
cg06372353	6.0E-02	1.3E-02	5.4E-06	4	147095635	LSM6 homolog, U6 small nuclear RNA associated (S. cerevisiae)
cg17922359	-5.8E-02	1.3E-02	8.4E-06	4	13530923	NA
cg12827530	-5.1E-02	1.1E-02	5.5E-06	5	80529121	NA
cg17483297	-8.2E-02	1.7E-02	1.2E-06	5	175084743	histamine receptor H2
cg19246007	-4.3E-02	9.1E-03	3.5E-06	6	56112216	collagen, type XXI, alpha 1
cg20548013	3.1E-02	6.7E-03	4.6E-06	6	12887210	phosphatase and actin regulator 1
cg22235018	-3.9E-02	8.7E-03	1.0E-05	6	50692150	transcription factor AP-2 delta (activating enhancer binding protein 2 delta)
cg24718382	3.8E-02	8.2E-03	4.7E-06	6	159462137	T-cell activation RhoGTPase activating protein
cg18325439	-8.2E-02	1.8E-02	3.8E-06	7	71217320	NA
cg21289191	3.2E-02	7.1E-03	7.2E-06	7	47694745	chromosome 7 open reading frame 65
cg11868900	-3.6E-02	7.9E-03	7.2E-06	8	49648035	EF-hand calcium binding domain 1
cg13434842	-4.5E-02	9.6E-03	3.6E-06	8	11567896	GATA binding protein 4

cg03330490	-7.3E-02	1.5E-02	2.4E-06	10	94452236	hematopoietically expressed homeobox
cg14924620	3.2E-02	7.0E-03	7.4E-06	10	50342114	family with sequence similarity 170, member B
cg24632480	4.3E-02	9.3E-03	5.9E-06	10	81864082	NA
cg18080819	3.7E-02	8.3E-03	8.1E-06	11	70505972	SH3 and multiple ankyrin repeat domains 2
cg27384476	3.5E-02	7.8E-03	9.9E-06	11	76367177	NA
cg01963134	-9.6E-02	2.1E-02	7.7E-06	12	39299364	copine VIII
cg02622052	-4.0E-02	8.8E-03	9.1E-06	12	41086274	contactin 1
cg04733537	-4.4E-02	9.6E-03	6.4E-06	12	130388748	transmembrane protein 132D
cg03848483	-3.7E-02	8.1E-03	6.9E-06	16	85969392	NA
cg09530163	-5.2E-02	1.1E-02	5.4E-06	16	55512822	matrix metallopeptidase 2 (gelatinase A, 72kDa gelatinase, 72kDa type IV collagenase)
cg10177032	-7.6E-02	1.7E-02	5.9E-06	16	4103492	adenylate cyclase 9
cg14073722	-8.1E-02	1.7E-02	1.8E-06	17	50235737	carbonic anhydrase X
cg17039236	-8.4E-02	1.9E-02	7.4E-06	18	77548049	NA
cg10358533	-6.4E-02	1.4E-02	7.4E-06	19	34114209	carbohydrate (N-acetylgalactosamine 4-0) sulfotransferase 8
cg14213992	-3.9E-02	8.6E-03	7.0E-06	19	40503358	zinc finger protein 546
cg17768491	-7.8E-02	1.7E-02	9.6E-06	19	41018876	spectrin, beta, non-erythrocytic 4
cg17936725	-4.2E-02	9.4E-03	8.8E-06	19	11925679	zinc finger protein 440
cg17971015	-5.3E-02	1.2E-02	7.7E-06	19	12306198	NA
cg26329715	-7.2E-02	1.5E-02	3.5E-06	20	61583987	solute carrier family 17, member 9

Grip Strength

CpG	Beta	SE	P	CHR	Position	Gene Name
-	-	-	-	-	-	-

Six Metre Walk Speed

CpG	Beta	SE	P	CHR	Position	Gene Name
cg04299400	-2.8E-02	5.7E-03	1.8E-06	1	240279726	formin 2
cg25319656	-9.8E-02	2.2E-02	6.4E-06	7	157933750	protein tyrosine phosphatase, receptor type, N polypeptide 2
cg05942562	-5.3E-02	1.0E-02	3.3E-07	8	8177911	homolog of rat pragma of Rnd2
cg23909079	-3.2E-02	7.2E-03	7.7E-06	10	87403707	glutamate receptor, ionotropic, delta 1
cg05248321	3.3E-02	6.8E-03	1.2E-06	14	20898128	kelch-like family member 33
cg21562740	-3.5E-02	7.5E-03	3.8E-06	15	78630426	NA
cg01130056	-6.3E-02	1.3E-02	2.1E-06	16	65528415	uncharacterized LOC283867
cg06614257	-5.3E-02	1.1E-02	3.9E-06	17	7758608	transmembrane protein 88
cg10508317	-4.4E-02	9.7E-03	6.9E-06	17	76355146	suppressor of cytokine signaling 3

cg15020801	2.5E-02	5.4E-03	4.3E-06	17	46022809	pyridoxamine 5'-phosphate oxidase
cg15416179	-6.7E-02	1.4E-02	1.3E-06	17	21189859	mitogen-activated protein kinase kinase 3

Supplementary Table 3: Associations between Hannum age acceleration at wave 1 and fitness variables adjusted for age and sex.

	Age acceleration		
	Beta*	SE	P
g_f †	-0.08	0.03	0.015
Grip Strength (kg)	-0.04	0.02	0.034
FEV_1 (l)‡	-0.008	0.02	0.72
6m Walk Time (s)	0.04	0.03	0.20

* Both age acceleration and the system integrity variables were standardised inputs to the regression analyses, implying that the beta values can be read as semi-partial correlations. Additional adjustments were also made for height (grip strength, FEV_1, and 6m walk) and smoking (FEV_1). †fluid type general intelligence ‡Forced Expiratory Volume in one second
